# Microstructure and Interfacial Reactions of Resistance Brazed Lap Joints between TC4 Titanium Alloy and 304 Stainless Steel Using Metal Powder Interlayers

**DOI:** 10.3390/ma14010180

**Published:** 2021-01-02

**Authors:** Pengxian Zhang, Zhizhong Fang, Shilong Li

**Affiliations:** 1 State Key Laboratory of Advanced Processing and Recycling of Nonferrous Metals, Lanzhou University of Technology, Lanzhou 730050, China; fangzhizhong1573@163.com (Z.F.); shilongli1990@163.com (S.L.); 2 School of Materials Science and Engineering, Lanzhou University of Technology, Lanzhou 730050, China

**Keywords:** resistance brazing, Ti-Fe intermetallic compounds (IMCs), metal powder layer, multi-reaction zone structure, metallurgical mechanism

## Abstract

In the brazing joint between titanium alloy and stainless steel, a lot of Fe-Ti intermetallic compounds (IMCs) can be easily formed to make joints crack. A lap resistance brazing process with metal powder layers on both sides of the filler metal was used to solve this problem. The microstructure and metallurgical behavior of joints was studied through comparative experiments. The result showed that Nb, V and Cr powders and the solder reacted with the base material to form a new phase, which replaced the Ti-Fe brittle phase in the joint. At the same time, metal powder clusters hindered the diffusion of Ti and Fe elements and improved the distribution of new phases. The established atomic reaction model revealed the metallurgical behavior and formation mechanism of the joints. Therefore, the intervening position of the metal powder layer and the multi-reaction zone structure are the main reasons the shear strength of joints is improved.

## 1. Introduction

The group weldment of the titanium alloy and stainless steel can make full use of the advantages of these two materials and have good economic benefits simultaneously, so its high-quality joints have extensive application prospects in nuclear, petrochemical, transportation and aerospace industries [1,2,3]. However, significant differences in the physical properties of these two dissimilar materials, such as their linear thermal expansion coefficient and melting point, can introduce considerable residual stress. Metallurgical incompatibility will result in the formation of brittle Ti-Fe series intermetallic compounds (IMCs), severely deteriorating the joint strength [4,5,6,7].

A rational solder is usually chosen as the middle transition layer to block the formation of brittle intermetallic compounds (IMCs) at the titanium alloy/stainless steel interface. Existing research indicates that welding the solid phase with the addition of an intermediate transition metal was an effective method to achieve a reliable connection between titanium alloy and stainless steel [4,8,9,10]. Parameters such as welding current, welding time and electrode pressure are important to consider in resistance spot welding. Qiu Ranfeng [11] et al. used a 0.1 mm thick niobium foil as an interlayer for resistance spot welding of titanium and Q235 mild steel sheets. The results showed that the niobium middle layer effectively hindered the formation of Fe-Ti intermetallic compounds (IMCs) in the joint under reasonable welding parameters, and the shear strength of the joint was significantly improved. Jung Gu Lee [12] et al. used a commercially available 72Ag–28Cu (wt%) alloy brazing filler to connect Ti and stainless steel. As a result, the Ag interlayer effectively prevented the diffusion of Ti from the base metal to the joint region, and no detrimental intermetallic phases were produced, which considerably improved the bonding strength of the joint. M.K. Lee [13] added a Cr/V/Ni composite metal foil layer and Ti-based amorphous solder between industrial pure titanium and stainless steel, and they heated the joints in an infrared manner. The joints formed a multilayered structure, and the brittle phases were generated less. Using multicomponent alloy brazing filler metal as a transition layer can significantly reduce the influence of the reaction interface on the mechanical properties. However, the composition of solder alloy used in the industry is still relatively simple, and they cannot meet the requirements to reliably connect titanium alloy/stainless steel. The composition and metallurgical behavior also need further study [14,15,16]. In particular, plenty of investigators [17,18] have put forward different methods to improve the microstructure and properties of joints, such as metal foils and electroplated layers as intermediate transition layers, for titanium alloy/stainless steel brazing connections. However, the addition of the metal layer artificially increased the interface, and the brittle phases tended to concentrate on the interface, causing the interface to become a weak point in the joint [19,20]. Therefore, how transition metal elements are involved is also a key factor influencing the reliable connection of titanium alloy/stainless steel.

In this study, titanium alloy and stainless steel resistance brazing using Cr, Nb and V powder layers and brazing filler metal as the intermediate transition layers was attempted. The influences of the metal powder layer intervention on the mechanical properties, interface morphology, microstructure and atomic diffusion behavior of the titanium alloy/stainless steel brazed joint were investigated. Furthermore, we explored the metallurgical reaction mechanism of the joint after the powder layer was applied, and the reasons the mechanical properties of the joint were improved.

## 2. Materials and Methods

**Figure 1:**
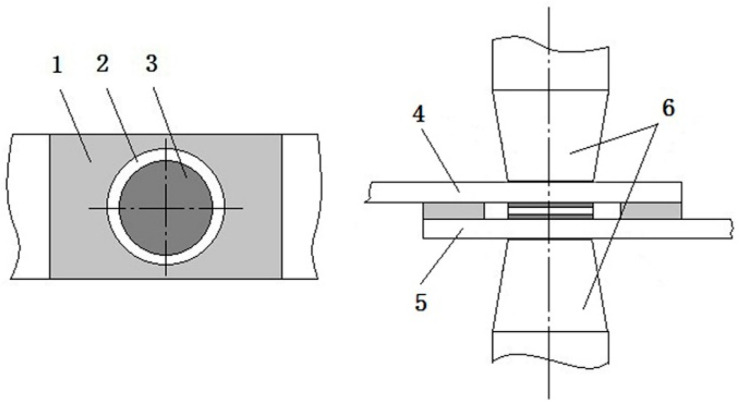
Diagram of joint assembly: (1) sealant; (2) reserved clearance; (3) metal powder and solder; (4) titanium alloy; (5) stainless steel; (6) electrode.

TC4 (Ti-6Al-4V) titanium alloy and 304 stainless steel (304SS) were used as the base metals and had the dimensions of 80 mm × 20 mm×1.5 mm. Their chemical composition and mechanical properties are listed in Table 1 and Table 2, respectively. The filler metal was a BAg45CuZn (45Ag-30Cu-24Zn-0.01Cd-0.05Si, wt%) silver base solder with a thickness of 0.2 mm. The metal powder comprised Cr, Nb and V powders with a 400 mesh particle size, and its purity was more than 99.9%. A single-component, ultrahigh temperature structural adhesive (YK-8907 1300 °C) was selected to prevent the joint from being oxidized in the resistance brazing process. The brazing filler metal was punched into a wafer with a diameter of 14 mm before the joint was assembled. The surfaces to be brazed were carefully ground and polished, and then they were ultrasonically cleaned in an acetone solution for more than 3 min and dried by air blowing. The joint assembly method is shown in Figure 1. First, a special mold was fixed on the surface of the stainless steel and titanium alloy, and a vibrating screen was used to spread Cr powder and Nb, V mixed powder evenly on the surface of the base material in area 3. Afterwards, the solder wafer was placed on the Cr powder layer and compacted under a pressure of 0.2 Mpa. Next, the sealant was smeared evenly on area 1 and held there for a moment. Finally, a titanium alloy base material with the Nb, V mixed powder layer was overlapped on the stainless steel adhesive layer and the solder layer according to the assembly positioning line, and this was pressed tightly and left standing for welding.

TC4 titanium alloy and 304 stainless steel plates overlapped for 35 mm along the length of the specimens, and the solder joint was in the center of the overlapping section. After resistance brazing, the tensile properties of the brazed joint were investigated using a universal testing machine (AG-10TA, SHIMADZU, Kyoto, Japan) at a test speed of 1 mm/min at room temperature. The orientation of the tensile loading was parallel to the joint interface. The test specimen was cut from the joint by electrical discharge machining for metallographic analysis. The cross-sections of the brazed joints were grounded, polished and then etched with a different reagent for separating materials. The structure of the joints was examined in cross-sections using both optical microscopy (OM, MeF3, LEICA, Wetzlar, Germany) and scanning electron microscopy (SEM, JSM-6700F, JEOL, Tokyo, Japan) coupled with energy-dispersive spectroscopy (EDS). The dispersion of elements across the joint interface was measured by X-ray line scans, and the phases and compositions in the joints were studied using X-ray diffraction (XRD, D8Advance, BRUKER, Karlsruhe, Germany).

To verify the influence of metal powders on the joint microstructure and mechanical properties, the two joint combinations shown in Table 3 were designed for titanium alloy/stainless steel resistance welding. By setting the welding current, welding time and other process parameters in resistance brazing, the microstructure and properties of the two types of joints were analyzed.

## 3. Results and Discussion

### 3.1. Effect of Powder Layer Intervention on Mechanical Properties of Joints

**Figure 2:**
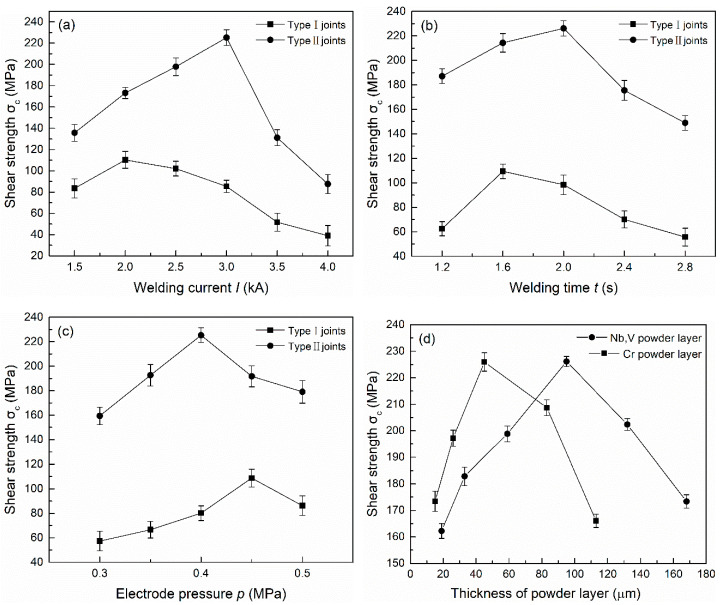
The influence of different process parameters on the shear strength of two types of joints: (**a**) welding current; (**b**) heating time; (**c**) electrode pressure; (**d**) thickness of powder layer (Type II).

The mechanical properties of the joints were assessed by the tensile test method, taking shear strength as the evaluation index. The welding current (*I*), the heating time (*t*), the electrode pressure (*p*), the thickness of the Cr powder layer (H1) and the mixed powder layer of Nb and V (H2) were used as the main process parameters. The experimental design was orthogonal with five factors and three levels. The mechanical properties of the two types of brazed joints were tested to explore the best process parameters. Figure 2 shows the influence of different process parameters on the shear strength of the two types of joints, and the best process parameters and the maximum shear strength (σ_c_) can be seen. The shear strength was averaged over three test results under the same conditions. Under the optimal process parameters, the shear strength of the joints with the powder layer was nearly double that of the joint without the additional layer, reaching 226 MPa. Therefore, the addition of the powder layer under reasonable parameters significantly improved the titanium alloy/stainless steel brazed joint shear strength.

### 3.2. Effect of Powder Layer Intervention on the Microstructure of Joints

**Figure 3:**
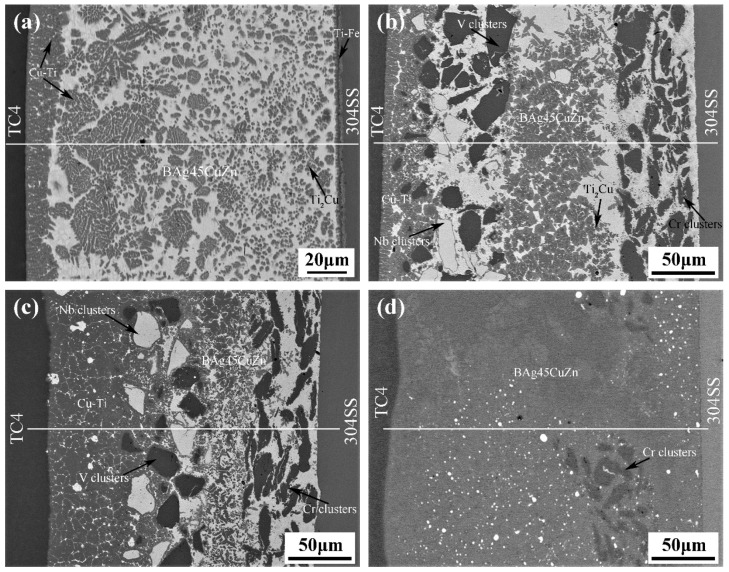
Microstructure of two types of joints under different welding currents: (**a**) 2 kA (Type I); (**b**) 2 kA (Type II); (**c**) 3 kA (Type II); (**d**) 4 kA (Type II).

**Figure 4:**
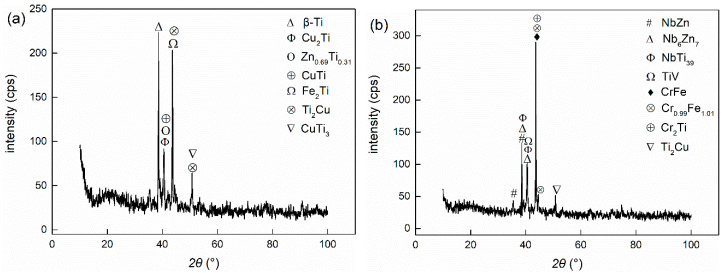
X-ray diffraction analyses of different brazing joints: (**a**) Type I (2 kA); (**b**) Type II (3 kA).

Through the characterization of the microstructure, it can be ascertained how the metal powder particles participated in the metallurgical reaction of the joints and the role they played in the joint component. SEM and EDS analyses were carried out in the central section of the welding zone in the Type I and Type II joints as the observed surface. The microstructure of joints under different welding currents is illustrated in Figure 3. The interface was divided into three zones: the left and right zones are TC4 titanium alloy and 304 stainless steel, respectively, and the middle is the brazing seam zone. XRD results of the joints are shown in Figure 4. The addition of powder layers changed the species and content of compounds in the joints. The brittle phases of Fe-Ti and Cu-Ti in joints were greatly reduced and replaced by Nb-Zn and Cr-Fe compounds as well as Ti-V and Ti-Nb solid solution phases.

In the process of titanium alloy/stainless steel resistance brazing, the welding current played a dominant role in the generation of resistance heat. The added intermediate layer underwent a metallurgical reaction with the base metal under the effect of resistance heat, which affected the formation of the joint microstructure. Figure 3a shows the microstructure of the Type I joint, and the welding current was 2 kA. The reaction zone was mainly composed of the brazing filler metal layer and two transition layers near the base metal. The transition layer near the titanium alloy side primarily consisted of grey Cu-Ti intermetallic compounds (IMCs). The solder layer was principally composed of white BAg45CuZn with a grey Cu-Ti brittle phase dispersed throughout. The transition layer near the stainless steel side was a hybrid structure with a large amount of Ti-Fe, a Cu-Ti brittle phase and a small amount of brazing filler metal. Therefore, the shear strength of the joints could not be enhanced.

Figure 3b–d shows the microstructure of Type II joints under different welding currents. The intervention of the metal powder layer made the Type II joint structure distribution more complicated. The most notable feature was the disappearance of the transition layer on the stainless steel side. The reaction zone was primarily composed of a Cr powder layer, a Nb and V mixed powder layer, a solder layer and the transition layer on the titanium alloy side. In Figure 3b, the metal powder and brazing material still maintained the original layer morphology under a welding current of 2 kA, and the transition layer near the titanium alloy side became narrower than the Type I. The size of black Cr, V particle clusters and white Nb particles were large in the powder layer, and the arrangement was relatively tight. The EDS result showed that the transition layer was principally composed of Cu-Ti intermetallic compounds (IMCs) and a small amount of Ti-Nb, Ti-V solid solution. The powder layer was mainly composed of metal particle clusters and unreacted solder. The brazing material layer was chiefly a mixed structure of Cu-Ti, Ti-Nb, Ti-V and other new phases and brazing material. As shown in Figure 3c, the width of the powder layer and the solder layer decreased, and the width of the transition layer near the titanium alloy increased accordingly when the welding current was 3 kA. The size of the particle clusters became significantly smaller in the powder layer and they were distributed around the newly born phase dispersedly. The boundary between the layers was no longer apparent. The content of the Ti-based solid solution in the transition layer near the titanium alloy increased, and since the Nb-Zn intermetallic compound was dispersed in the solid solution, the content of the Cu-Ti brittle phase decreased accordingly. When the welding current was further increased to 4 kA, as shown in Figure 3d, the Nb and V mixed powder layer and the solder layer in the joint completely disappeared, and the Cr powder layer was greatly decreased. As a result, plenty of Fe-Ti intermetallic compounds (IMCs) also appeared in the entire joint structure, except for the formation of Ti-based solid solution and the brittle phases of Cu-Ti and Nb-Zn, which was the main reason the shear strength of the joints decreased [21,22].

We analyzed the microstructure of the joint under different welding currents. It can be seen the resistance heat was insufficient when the welding current was small; therefore, added metal powder has a limited metallurgical reaction and was still present in the joint structure in the form of powder clusters. The degree of atom diffusion was also lower, so the shear strength of joints was low. With an increase in welding current, the Nb and V mixed powders combined with diffused Ti atoms to form a solid solution, and a hybrid structure was formed with the brazing material that was not involved in the reaction. A considerable amount of Cr powder on the stainless steel side still existed in the form of powder clusters, which blocked the diffusion of Fe. Therefore, the shear strength of the joint was high. The resistance heat of joints was too large, and the Nb, V mixed powder completely disappeared when the welding current was larger. The metallurgical reaction was violent, and the Cr powder clusters disappeared in larger quantities. The degree of atomic diffusion was bigger that attributed to a significant reduction in the blocking effect of the interlayer on the diffusion of Ti and Fe atoms. A large number of brittle phases appeared in the joint, which significantly decreased its strength. Therefore, the added metal powder effectively prevented the diffusion of Ti and Fe elements only under the appropriate welding current. Additionally, a large amount of solid solution was formed in the joint, and a better performing brazed joint was obtained.

### 3.3. Effect of Powder Layer Intervention on Atomic Diffusion Behaviour of Joints

**Figure 5:**
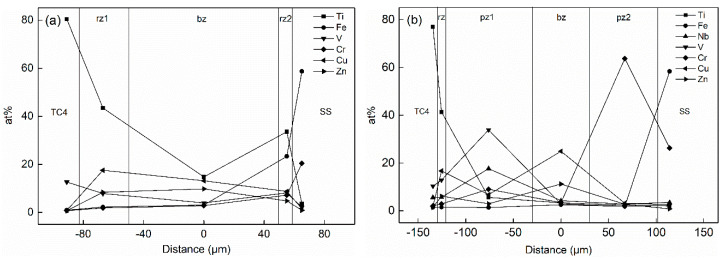

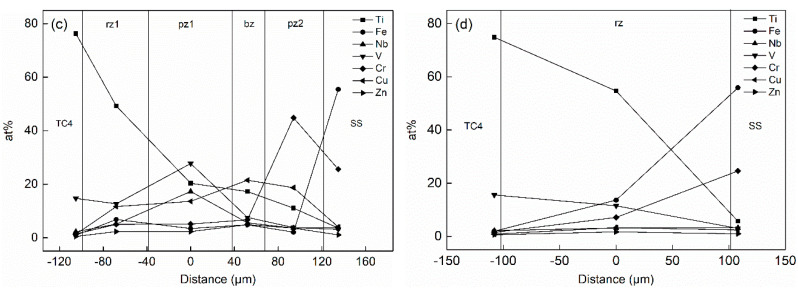
Atomic concentration changes of two types of joints under different welding currents: (**a**) 2 kA (Type I); (**b**) 2 kA (Type II); (**c**) 3 kA (Type II); (**d**) 4 kA (Type II).

The concentration distribution of Ti and Fe elements in the joint played a critical role in the formation of intermetallic compounds (IMCs), solid solutions and other microstructures in the titanium alloy/stainless steel resistance brazing process. The effect of the metal powder layer on atom-diffusion was reflected in the atomic concentration distribution measured by EDS. Figure 5 shows the variation curve for the atomic concentration of Ti, Fe and other principal elements with the diffusion distance in the weld region of the two types of joints. SS and TC4 are regions of stainless steel and titanium alloy base material. pz1 and pz2 are the Nb, V mixed powder and the Cr powder areas. bz is the brazing material reaction area. rz is the reaction zone of the joint. rz1 and rz2 are the transition zones on titanium alloy side and the stainless steel side, respectively. Figure 5a, c shows the atomic concentration distribution curves of the Type I and Type II joints under welding currents of 2 kA and 3 kA, respectively. The content of Ti in the rz1 area increased from 43% in the Type I joint to 49% in the Type II joint. The content of Ti and Fe in the area near the stainless steel decreased from 34% to 23% in the Type I joint and from 11% to 2% in the Type II joint, respectively. The existence of the Nb and V metal powder layer significantly increased the degree of Ti element diffusion, many Ti elements were consumed to form intermetallic compounds (IMCs). Moreover, the presence of the Cr powder layer formed the Cr-rich region in the rz2 zone, which blocked the opposite diffusion of Ti and Fe elements and inhibited the formation of brittle phases.

The atomic concentration distribution curves of the Type II joint with welding currents of 2, 3 and 4 kA are shown in Figure 5b–d. As the welding current increased, the amount of metal powder participating in the reaction increased, and its element content decreased. Meanwhile, the hindrance function of the metal powder gradually reduced, and the degree of diffusion of Ti and Fe elements increased. As shown in Figure 5b, the contents of Ti, Nb and V in the pz1 area were 6%, 18% and 34% when the welding current was 2 kA, respectively. The content of Cr in pz2 was 74%. The content of Fe was less than 3% in the entire joint area. As shown in Figure 5c, the content of Ti increased to 20% in the pz1 region when the welding current increased to 3 kA, and the content of Fe reached 6% in the rz1 zone. The content of Nb and V elements in pz1 decreased to 17% and 28%, and the content of Cr in pz2 reduced to 45%. As shown in Figure 5d, the powder layer disappeared when the welding current increased to 4 kA. The contents of Ti and Fe in the entire joint were 55% and 14%, and the contents of Nb, V and Cr decreased to 3%, 12% and 7%, respectively. Therefore, the blocking effect of the powder layer was remarkable when the welding current was small. Thermogenesis at the joint increased as the current increased, and more powder participated in the metallurgical reaction, which made the originally clear layer structure in the joint gradually become blurred. After the powder layer disappeared, Ti and Fe freely diffused and formed a brittle phase in the joint, which deteriorated the structure.

### 3.4. Effect of Powder Layer Intervention on Metallurgical Behaviour of Joints

**Figure 6:**
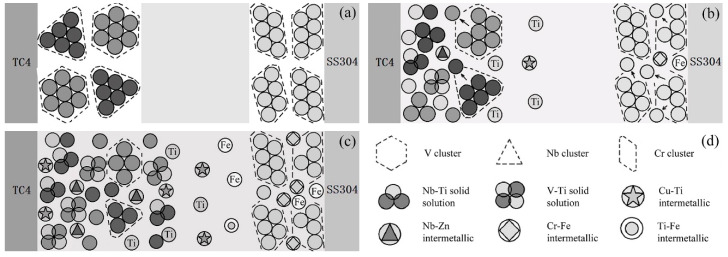
Metallurgical reaction process of joints with the participation of metal powder particles: (**a**) before brazing; (**b**) during brazing process; (**c**) after brazing; and (**d**) schematic of each substance.

In order to characterize the influence of metal powder on the metallurgical behavior of the joint, metal powder particles were regarded as polyatomic clusters, and a metallurgical reaction model was established. Figure 6 shows the metallurgical reaction process of the metal powder particles involved in the joint. Figure 6d shows the identification of each substance. Brazing is the wetting and spreading of molten solder on the surface of a solid base material, and then metallurgical processes such as dissolution, atomic reaction and diffusion occur. When resistance brazing is performed after a powder is inserted, under the action of heat and pressure, the molten solder spreads on the surface of the base material after passing through the gap of the powder layer. Since atoms can diffuse easier in the liquid state, the base material atoms will diffuse and migrate into the brazing material between the metal powder particles, increasing the concentration of the base material atoms in the brazing material. Before the reaction, each substance showed a stable atomic structure, as shown in Figure 6a. During the reaction, resistance heat comes from internal heat sources, so the metal particles generate heat themselves, and the energy of the atomic cluster increases. The atoms at the edge first break away from the atomic clusters and then enter the liquid solder to combine with a large number of Ti atoms. Corresponding compounds were generated in the cluster gaps, as shown in Figure 6b. As the reaction progressed, the atoms on the edge of the cluster continued to enter the liquid solder, and the metal powder was continuously consumed in a peeling reaction. As shown in Figure 6c, the metal particles that were closer to the base material first reacted with the base material atoms, and their edges gradually became smooth and generated corresponding compounds. Additionally, the metal particles far away from the base material still existed in the solder, blocking the diffusion and migration of the base material atoms. On the one hand, when the metal powder intervened in the titanium alloy/stainless steel brazing joint, diffusion of the base material atoms to the opposite side was blocked. On the other hand, the morphological structure of the reactant changed due to the changing atomic reaction.

The titanium alloy/stainless steel brazed joint showed a multi-layer structure after the metal powder intervened, and the diffusion degree of Ti and Fe elements increased. Some of the powder participated in the metallurgical reactions and consumed plenty of Ti and Fe elements. The diffusion of Ti and Fe to the opposite side was effectively hindered by the powders not involved in the reaction and reduced the number of Ti-Fe brittle phases. Meanwhile, the existence of powder particles changed the morphology of the reaction interface. The metallurgical reaction was performed on the surface of the powder particles. Therefore, the brittle phases dispersed and played a role of the second-phase strengthening for joints. Additionally, the shear strength of joints improved due to the formation of solid solution phases.

## 4. Conclusions

Cr, Nb and V powder interlayers were applied to conduct resistance brazing between Ti-6Al-4V titanium alloy and 304 stainless steel. The atomic reaction model characterized the formation mechanism and metallurgical behavior of the joints in the brazing process. The main results are listed as follows:

(1) The intervention of Cr, Nb and V powders changed the microstructure of the titanium alloy/stainless steel brazed joint. The metal powder and the solder together formed the multi-reaction microstructure zone, the diffusion behavior of the elements was affected, the content of the Ti-Fe brittle phase was reduced, and the new brittle phase was dispersed in the solid solution structure of the joints. This is a reason for improving joint shear strength.
(2) The addition of metal powder layer changed the metallurgical behavior of the joint. The existence of metal particles changed the morphology of the reaction interface, and the metallurgical reaction concentrated on the surface of powder particles, showing a peeling type reaction, which also altered the existence form of the new phase in the joint. The powder involved in the reaction consumed a large amount of Ti and Fe elements, while those that did not react continued to play a hindering role. This is another reason to improve the mechanical properties of titanium alloy/stainless steel brazed joints.

## Figures and Tables

**Table 1:** Chemical composition of base metals (wt%).

Alloy	Fe	Cr	Ni	Al	Ti	V	Mn	Si	C
TC4	0.30	0	0	6.15	Bal.	4.20	0	0.07	0.10
304SS	Bal.	18.83	10.26	0	0	0	2	0.62	0.07

**Table 2:** Physical properties of base metals.

Alloy	Density, g/cm^3^	Melting Point, °C	Expansion Coefficient, ×10^−6^ K^−1^	Tensile Strength, MPa
TC4	4.55	1660	7.14	895
304SS	7.93	1398~1454	18.4	563

**Table 3:** The combination mode of the brazing joint.

Types of Brazing Joints	Combination Mode of the Brazing Joint	Mass Ratio of Mixed Powder
Type I	TC4 + solder + 304SS	-
Type II	TC4 + (Nb,V) + solder + Cr + 304SS	m(Nb):m(V) = 17:33

## Data Availability

Data sharing not applicable.
